# Phylogenetic Analysis of Belgian Small Ruminant Lentiviruses Supports Cross Species Virus Transmission and Identifies New Subtype B5 Strains

**DOI:** 10.3390/pathogens9030183

**Published:** 2020-03-03

**Authors:** Rodolphe Michiels, Nadjah Radia Adjadj, Nick De Regge

**Affiliations:** Unit of Enzootic, Vector-Borne and Bee Diseases, Sciensano, Groeselenberg 99, 1180 Brussels, Belgium; nadjahradia.adjadj@sciensano.be (N.R.A.); nick.deregge@sciensano.be (N.D.R.)

**Keywords:** Maedi-Visna virus, caprine arthritis encephalitis virus, small ruminant lentivirus, phylogeny, Belgium, cross-species transmission, subtype B5

## Abstract

Small ruminant lentiviruses (SRLV) are a group of highly divergent viruses responsible for global and fatal infections in sheep and goats. Since the current phylogenetic classification of these viruses was proposed in 2004, it nowadays consists out of 5 genotypes and 28 subtypes. In support of our national SRLV control program, we performed the genetic characterization of SRLV strains circulating in the Belgian sheep and goat population. Fourteen sheep and 9 goat strains were sequenced in the *gag-pol* and *pol* regions using the method described by Shah. Most SRLV strains from sheep and goats belonged to prototype A1 and B1 subtypes, respectively. We, however, also found indications for cross-species transmission of SRLV strains between sheep and goats and vice versa, and identified a new subtype designated as B5. An in-depth analysis of the current SRLV phylogeny revealed that many subtypes have been defined over the years based on limited sequence information. To keep phylogeny as a useful tool, we advocate to apply more rigorous sequencing standards to ensure the correct classification of current and new emerging strains. The genetic characterization of Belgian SRLV strains will help in the development of appropriate diagnostic tools to assist the national control program.

## 1. Introduction

Maedi-Visna virus (MVV) and caprine arthritis encephalitis virus (CAEV), also referred to as small ruminant lentiviruses (SRLV), are two related retroviruses infecting sheep and goats [1,2]. They are both responsible for a persistent and lifelong infection by targeting the monocytes of the host and the stem cells located in the bone marrow [2]. SRLV induce a multisystemic disease with progressive inflammatory lesions in the mammary gland, lungs, joints, and the brain. Symptoms such as pneumonia, arthritis, and mastitis are commonly observed in one third of the infected animals [3,4]. SRLV are mainly transmitted vertically to newborns via the ingestion of infected milk and colostrum but also horizontal transmission can occur at any age via the inhalation of viral particles between animals housed in close contact [5,6]. The incidence of these infections causes considerable economical losses in animal production and no therapy or vaccine is currently available [7].

Initially, MVV in sheep and CAEV in goats were described to be strictly host specific. Phylogenetic studies have however revealed that cross-species transmission has occurred in the past. For this reason, both viruses are nowadays referred to as one group called SRLVs [1,7,8]. In 2004, Shah et al. proposed a classification of SRLV strains based on sequence information of 2 genomic regions, the *gag-pol* (1.8 kb) and the *pol* (1.2 kb) region. The *gag* gene encodes for three proteins, being the matrix (MA), nucleocapsid (NC), and capsid (CA) proteins, and the *pol* gene encodes for enzymatic proteins including the reverse transcriptase (RT) [9]. Sequencing of both regions resulted in an initial classification of SRLVs into four genotypes from A to D and further subdivision of genotype A and B strains into A1 to A7 and B1 to B2 subtypes [7]. Genotypes A and B strains were referred to as MVV-like and CAEV-like, respectively, and are the most predominant strains around the world. Group C strains were identified in Norwegian sheep and goats while genotype D strains comprised only a few strains from sheep and goats located in Switzerland and Spain [7,10].

Since this initial classification, many other strains have been characterized and added to the phylogenetic tree, but often based on much shorter genomic fragments than those proposed by Shah et al. [7,11]. This leads to a current SRLV phylogeny containing 5 genotypes and 28 subtypes. The high number of subtypes reflects the high genetic heterogeneity between SRLV strains, which in turn impairs the performance of diagnostic tests [12,13].

In Belgium, a recent nationwide seroprevalence study confirmed the presence of SRLV in 13% and 17% of sheep and goat farms, respectively [14]. Therefore, we decided to perform a genetic characterization of SRLV strains circulating in the Belgian sheep and goat population, based on the genomic fragments proposed by Shah et al. We also carried out a critical evaluation of the current SRLV classification and propose to apply more rigorous sequencing standards to ensure a correct classification of current and future SRLV subtypes.

## 2. Results

### 2.1. Analysis of the Current SRLV Phylogeny

Table 1 provides an overview of the fragment lengths and target regions that have been used to add new strains and identify new subtypes to the current SRLV phylogeny. 

The basis of the current classification of SRLV strains was proposed in 2004 by Shah et al. using the genetic sequence of a 1.8 kb fragment in the *gag-pol* region, a 1.2 kb fragment in the *pol* region, and a smaller fragment (279 bp) within the reverse transcriptase (RT) region. Subtypes A1 to A5 were classified based on sequence information in these three regions, just as subtypes B1, B2, and genotype C. Subtypes A6, A7, and genotype D were classified on the basis of the nucleotide sequence of only one of these regions. 

Later on, more strains were added to the phylogeny by others, but often based on the sequence of only a smaller part of one of the fragments initially proposed by Shah et al. This was the case for A8, A9, A10, A11, A18, B4, and genotype E which includes subtypes E1 and E2. Other studies also used smaller fragments but confirmed their classification in multiple regions. The identification of A12 and A13 was, for example, supported by the classification of 3 small fragments within the *env* and *gag* genes. 

This overview clearly shows that different fragments and regions have been used over time to characterize new strains. Since the classification of Shah et al., one new genotype and 17 new subtypes have been added to the current phylogeny but mostly based on fewer same sequence information as proposed in the initial classification. This raises the question whether the current classification with all the published subtypes would still hold when those strains would have been added to the phylogeny based on the full fragments proposed by Shah et al. Furthermore, different publications used different programs and methods to obtain their phylogenetic trees, what could further impact and complicate the comparison between studies.

### 2.2. Phylogeny of Belgian SRLV Strains in the Gag-Pol Region

Proviral DNA of 14 SRLV strains from sheep and 7 SRLV strains from goats originating from different Belgian provinces were successfully sequenced in the *gag-pol* region (Table 2), while no sequences were obtained in this region for the 1 SRLV strain present in sheep and 3 strains present in goats.

**Figure 1:**
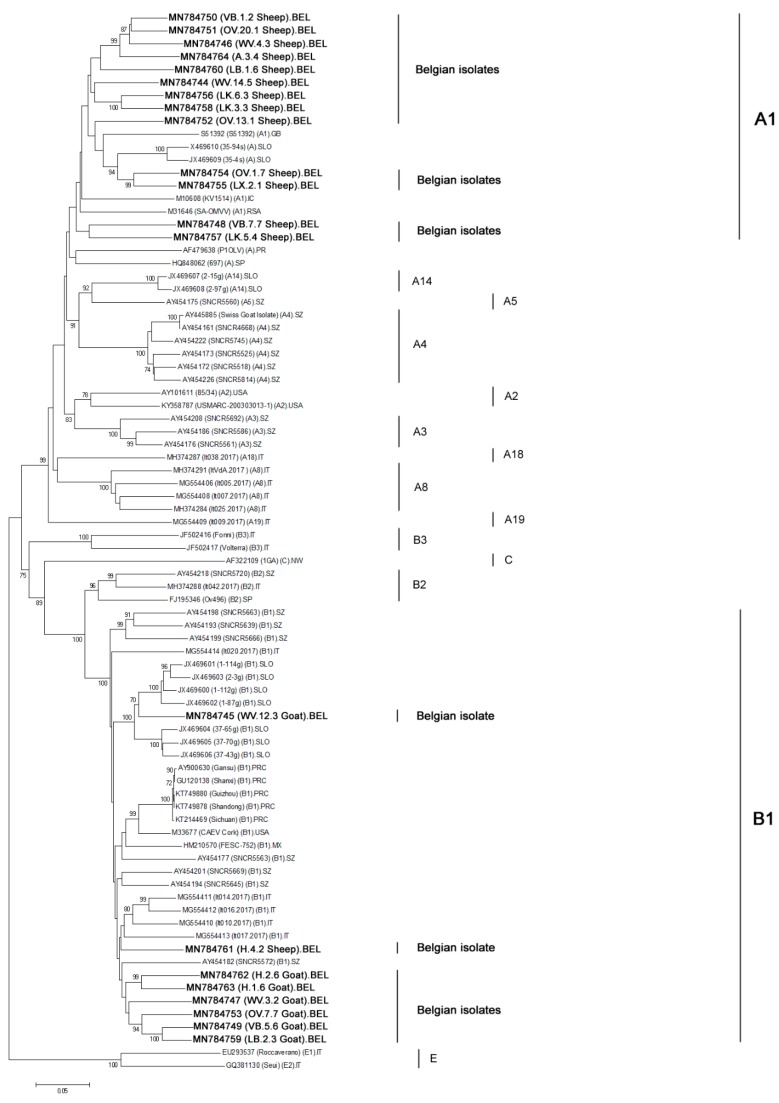
Phylogenetic tree was inferred by neighbor-joining using a 1516 bp consensus fragment located in the *gag-pol* region (1.8 kb). Only bootstrap values ≥70 are shown. All sequences are indicated by their GeneBank accession number and their names between brackets. For the Belgian isolates (21), indicated in bold, the provinces of origin are designated by their abbreviations: A: Antwerpen; H: Henegouwen; LB: Limburg; LK: Luik; LX: Luxemburg; OV: Oost-Vlaanderen; VB: Vlaams Brabant; WV: West-Vlaanderen. For the reference strains described in the literature, their subtype affiliations are named in parenthesis. For few strains, only the genotype affiliations were indicated. Country abbreviations are: BEL: Belgium; CA; Canada; FIN: Finland; GB: Great Britain; IC: Iceland; IT: Italy; MX: Mexico; NW: Norway; PR: Portugal; PRC: People’s Republic of China; RSA: Republic of South Africa; SLO: Slovenia; SP: Spain; SZ: Switzerland; USA; United States of America.

After alignment with sequences available in GeneBank, a phylogenetic analysis was performed using the neighbor-joining method on the consensus fragment of 1516 bp. Our analysis showed that 13 out of 14 sequenced Belgian SRLV strains from sheep belonged to subtype A1 (Figure 1; Table 2). A mean nucleotide diversity of 15.7% was identified between the reference strain KV1514 (A1) and the group of sheep strains associated to A1. Interestingly, one sheep strain (H.4.2) clustered within subtype B1 and is thus indicative for the occurrence of natural SRLV cross-species transmission from goats to sheep. This B1 strain showed a nucleotide distance of 10.9% to the reference B1 Cork strain. 

The 7 SRLV strains present in Belgian goats all showed to belong to subtype B1 in the *gag-pol* phylogeny (Figure 1; Table 2). They showed a nucleotide diversity of 12.5% with the Cork reference strain. 

To evaluate the robustness of our analysis, we alternatively performed the phylogeny also using the maximum-likelihood and the Bayesian inference method, which resulted in the same classification of all strains (data not shown).

When analyzing the phylogeny based on the *gag-pol* region, it is furthermore interesting to observe that the Norwegian strain (1GA; AF322109), that was considered to be a genotype C strain by Shah [7], is classified as a genotype B strain in the *gag-pol* region. Subtype E1 and E2 strains could also be incorporated in the phylogenetic tree since they have been fully sequenced after their first classification. When considering the whole *gag-pol* region, they also remain classified as a separate genotype.

### 2.3. Phylogeny of Belgian SRLV Strains in the Pol Region

**Figure 2:**
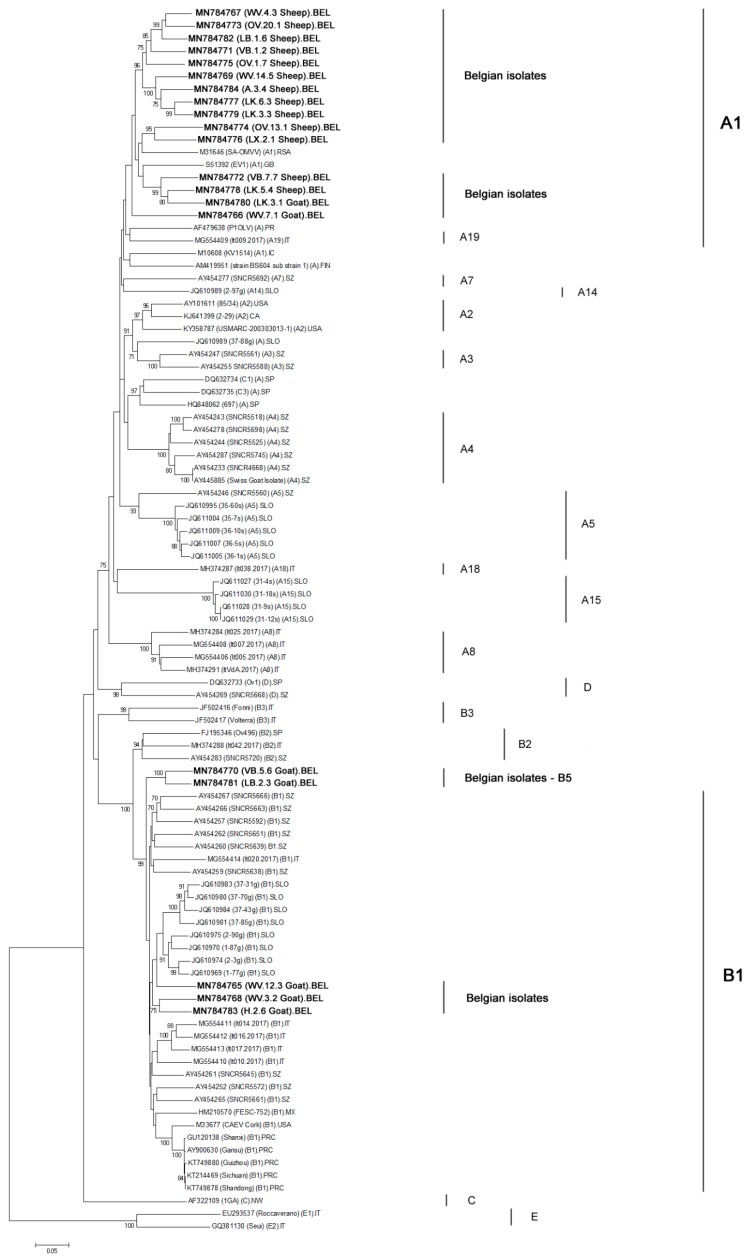
Phylogenetic tree was inferred by neighbor-joining using a 1068 bp consensus fragment located in the *pol* region (1.2 kb). Only bootstrap values ≥ 70 are shown. All sequences are indicated by their GeneBank accession number and their names between brackets. For the Belgian isolates (20), indicated in bold, the provinces of origin are designated by their abbreviations: A: Antwerpen; H: Henegouwen; LB: Limburg; LK: Luik; LX: Luxemburg; OV: Oost-Vlaanderen; VB: Vlaams Brabant; WV: West-Vlaanderen. For the reference strains described in the literature, their subtype affiliations are named in parenthesis. For few strains, only the genotype affiliations were indicated. Country abbreviations are: BEL: Belgium; CA; Canada; FIN: Finland; GB: Great Britain; IC: Iceland; IT: Italy; MX: Mexico; NW: Norway; PR: Portugal; PRC: People’s Republic of China; RSA: Republic of South Africa; SLO: Slovenia; SP: Spain; SZ: Switzerland; USA; United States of America.

Amplicons of 13 sheep strains and 7 goat strains were successfully obtained in the *pol* region proposed by Shah et al. (Table 2). The phylogenetic analysis based on a 1068 bp fragment showed that all SRLV strains from sheep clustered in subtype A1. Their mean diversity with the Icelandic reference strain KV1514 was 17.8% (Figure 2). No sequence for the *pol* fragment could be obtained for the SRLV strain from sheep (H.4.2) that clustered with genotype B strains in the *gag-pol* region. 

Five goat strains sequenced in the *pol* region belonged to genotype B (Figure 2) with a mean diversity of 12.9% compared to the reference Cork strain. These 5 Belgian goat isolates belonged to 2 different subtypes. Three strains (WV.12.3, WV.3.2, H.2.6) belonged to subtype B1 while two others (VB.5.6 and LB.2.3) formed a new separate cluster within genotype B (bootstrap value of 100%). We propose to designate them as subtype B5 strains. These B5 strains show a genetic diversity of 13.22% and 13.12% with the B1 reference Cork strain. A recombination analysis with all strains present in the phylogeny was performed to verify if indications could be found that the B5 strains resulted from a recombination of already described sequences in the *pol* region. No event of recombination between known strains was identified in the *pol* region. It is important to notice that both B5 strains also clustered together in the *gag-pol* region but belonged to subtype B1 in that fragment (see higher). The finding that strains can cluster to different subtypes depending on the fragment that is considered was also observed for the current subtype A19 strain. This strain namely belongs to subtype A19 only in the *gag-pol* region while it clusters with subtype A1 strains in the *pol* region (Figure 2). 

Two other SRLV strains from goats clustered within subtype A1, thereby providing evidence that SRLV transmission has occurred from sheep to goats as well. No sequences in the *gag-pol* region could be obtained for both strains (LK.3.1; WV.7.1) (Table 2).

The same classification was obtained in the *pol* region when we performed the maximum-likelihood and the Bayesian inference methods (data not shown).

Similar as mentioned already for the *gag-pol* analysis, subtypes E1 and E2 strains also form a separate genotype in the phylogenetic tree based on their sequences in the *pol* region.

## 3. Discussion

The current SRLV phylogeny consisting of 5 genotypes, which are further divided in multiple subtypes, emphasizes the high genetic variation that exists among SRLV strains. This high genetic diversity between strains often poses challenges for countries that implemented SRLV eradication programs since none of the existing diagnostic tests are capable to detect all circulating strains [13,24]. Since a voluntary SRLV eradication program is running in Belgium, we wanted to get a better insight in the SRLV strains circulating in our country and decided to perform a genetic characterization of Belgian SRLV strains present in naturally infected sheep and goats.

The method described by Shah et al. [7] was used for this work, meaning that we attempted to sequence the *gag-pol* and *pol* regions. These have been described to be conserved genomic regions and are widely accepted for SRLV classification [9,25]. We successfully obtained sequences in at least one of both regions for 14 out of 15 SRLV strains from sheep and 7 out of 10 SRLV strains from goats. The fact that no sequences could be obtained for all strains or fragments is most probably due to the low proviral load in infected animals and the high genetic heterogeneity of SRLV strains. This heterogeneity has been reported to be higher among isolates from goats than from sheep and this seems to be reflected in our results [13]. Several mechanisms could explain the high genetic variations existing between SRLV. The first and most important is mostly attributed to the lack of proofreading driven by the reverse transcriptase enzyme (RT). The low fidelity of the enzyme during the reverse transcription stage is responsible for the introduction of new mutations in the SRLV genome (0.2–2 mutations per genome cycle). A second origin of genetic variation can be related to the APOBEC3 enzyme, an intrinsic protein known to incorporate deleterious mutations into the viral genome by cytosine to uracil deamination. The excess of uracil in the reversely transcribed negative DNA strand can afterward lead to G-to-A mutations in the plus strand of proviral DNA. Along with this, macrophages, known to be SRLV target cells, are known to contain a higher amount of intracellular dUTPs. This excess of uracil can be incorporated into the DNA leading to more mutations in newly produced virions (reviewed in [13]). A third source of genetic evolution in lentiviruses that adds to their adaptability and genetic diversity is the occurrence of recombination between strains [26]. This mechanism allows viruses to combine genetic information which can lead to new emerging strains. Such events mostly occur when an animal is co-infected by two or more SRLV strains after cross or interspecies transmission [27]. The importation of infected animals and the lack of veterinary controls is also a another source of genetic heterogeneity within SRLV since new strains can be introduced into local herds [7].

Besides the expected finding of genotype A strains in sheep and genotype B strains in goats, we also found the presence of 2 genotype A1 strains in goats and one genotype B1 strain in a sheep. This indicates that cross species transmission from sheep to goats and vice versa has occurred. Cross species transmission of SRLV strains has been reported before [8,27,28] and such transmission events probably occur via transmission of viral particles via the air when different species are in close contact or via feeding of contaminated milk from sheep to goat or vice versa [8,26,29]. Although animal WV.7.1 came from a farm where both sheep and goats were present, our available information does not allow to deduce whether the cross-species transmission has occurred at this farm, or that this strain entered the farm via the purchase of an animal infected with an A1 strain. 

Remarkably, we could only obtain sequence information in either the *gag-pol* or the *pol* region using our methodology for these three strains indicative for cross species transmission. This suggests an even higher genetic divergence of these strains. This could be due to the fact that viruses are forced to adapt and evolve more rapidly when introduced into a new species, often resulting in the emergence of new diverging strains [26,30]. Some field studies have demonstrated such SRLV genetic adaptation induced by cross species transmission. Erhouma et al. showed that important genetic differences were present in the LTR region of proviral SRLV sequences after transmission from domestic goats to wild ibexes via natural contact. The *gag* gene, in turn, was better conserved [31]. Other studies also reported minor modifications in the genome after the passage of SRLV from sheep to goats and vice-versa [7,8,28]. In addition to this genetic adaptation to a new host, it could also be that these strains are the result of a recombination event that occurred in dually infected animals [26].

During analysis of the presented phylogenetic trees, it can be observed that the classification of strains to a specific subgroup can differ depending on the fragment that is analyzed. In the *gag-pol* phylogenetic tree, the strain belonging to subtype A19 is clearly separated from other genotype A subtypes [19], while in the *pol* tree, it clusters within subtype A1. A similar observation can be made when looking at the reference strains known to belong to genotype C. In the phylogeny based on the *pol* fragment, these indeed form a separate cluster, but when looking at the *gag-pol* sequence, they would be classified as genotype B strains. A similar observation was already made by Olech et al. who classified the genotype C strains within genotype B after sequence analysis of smaller fragments in the *gag* region. Kuhar et al., on the other hand, found the genotype C strains to form separate groups in both phylogenetic trees based on longer *gag-pol* and *pol* sequences but not as long as seen in this study [18,19]. A similar observation is made for some of our Belgian strains. Two epidemiologically unrelated strains form a separate cluster within genotype B when looking at the phylogenetic tree based on the *pol* fragment. We propose to designate them as genotype B5 strains, seeing the high bootstrap value of their cluster and the high genetic distance they have with others genotype B reference strains including B1, B2, and B3 (mean nucleotide distance of 17.9% in the *gag-pol* fragment and 18% in the pol fragment). In the *gag-pol* analysis, however, these strains cluster within the B1 subgroup. We therefore hypothesize that this strain finds its origin in a recombination between a B1 strain and an until hereto undescribed B5 strain upon co-infection of a goat. This seems more likely than a B1 strain accumulated by so many mutations in the *pol* genomic region.

Finally, analysis of the current phylogeny showed that many new genotypes and subtypes have been claimed based on shorter fragments than those proposed by Shah et al. It is not likely that the current phylogeny would stand if longer fragments would have been used. We therefore believe that more rigorous standards should be followed when adding strains to the phylogeny to ensure the correct classification of new emerging strains and suggest to use at least the *gag-pol* and the *pol* fragments proposed by Shah et al. Since phylogenetic analyses are a helpful tool to understand shortcomings of diagnostic tests, the availability of sufficiently long sequences will also be helpful to continuously improve diagnostic tests. Accessible sequence information for the *gag-pol* and *pol* region can help to develop and/or improve PCR tests for molecular SRLV detection. Furthermore, sequence information in the *gag-pol* region, covering epitopes of the capsid protein that are sometimes used as antigens in SRLV ELISAs [24], can also help to identify why some SRLV infected animals are not recognized by currently used ELISA tests and provide the necessary information to construct strain-specific ELISAs using strain-specific epitopes. Proof of concept studies on the use of peptides from SRLV SU5 and transmembrane proteins was already described for the detection of specific Spanish strains that were not detected by other ELISAs [32,33].

## 4. Materials and Methods 

### 4.1. Sample Selection

Samples used in this study originated from a previous study carried out to determine the SRLV seroprevalence in Belgium (Table 2). Samples came from both small-scale hobbyist farms and professional farms that were not involved in the SRLV national control program. Independent of the farm size, 7 animals were sampled per farm. More details on the methodology and sample collection can be found in Michiels et al. [14] and details on the samples used in this study are presented in Table 2. Fifteen out of 87 sheep herds and 10 out of 76 goat herds were found to contain at least one SRLV infected animal. In the current study, we aimed to characterize the SRLV strain present at each seropositive sheep and goat farm. For every positive farm, we selected the sample that showed to contain the highest amount of SRLV proviral DNA, i.e., the animal that showed the lowest Ct value in previously described qPCR tests [24,34] (Table 2). If none of the animals in the farm tested positive in qPCR, but were positive in serology, the animal with most SRLV specific antibodies (based on S/P value) was selected. Animals that appeared to be infected by both a genotype A and B strain based on the qPCR analysis in the previous study were omitted from the analysis [24].

### 4.2. DNA Extraction, PCR Amplification, and Sequencing

Leucocyte pellets were obtained by treating 1.5 mL of EDTA blood with 8.5 mL of hemolysis buffer (16.6 g NH_4_Cl, 2.0 g NaHCO_3_, 0.185 g diNa EDTA per L H_2_O; pH 7.4) [35]. After 20 min of incubation at room temperature, samples were centrifuged for 10 min at 3000× *g* and the pellet was resuspended in 200 μL of PBS. Genomic DNA, including proviral DNA, was then extracted from the leucocyte pellets using the QIAamp DNA Minikit (Qiagen, Hilden, Germany) following the manufacturer’s instructions. Purified DNA was eluted in 100 µL of elution buffer and stored at −80 °C until use.

We attempted to sequence all SRLV strains in the 1.8 kb *gag-pol* and the 1.2 kb *pol* region using the method of Shah et al. [7]. Different combinations of primers were used to obtain DNA fragments from the targeted regions. The 1.8 kb *gag-pol* region was amplified with the combination of P39-P37 primers, followed by nested PCRs using either P21-P41 or P40-P27. For few samples, P40-P37 was used instead of P40-P27. The amplification of the 1.2 kb *pol* region was done using the combination of P28 and P32 followed by a nested PCR using P29-P35 primers. 

Amplification with P39-P37 and P28-P32 was done as follows: activation of the FastStart Taq DNA polymerase (Roche, Basel, Switzerland) at 95 °C for 15 min, 45 cycles of denaturation at 94 °C for 30 s, annealing at 55 °C for 1 min, and extension at 72 °C for 2 min. For the nested PCRs, the following cycling conditions were used: 95 °C for 15 min, followed by 45 cycles of denaturation at 94 °C for 30 s, annealing for 1 min at 51 °C for P40-P27 and P40-P37, 53 °C for P21-P41, and 57 °C for P29-P35, followed by an extension step of 1 min at 72 °C for P21-P41 or 2 min for the other primer combination. The final reaction volume consisted on 25 µL of 2× Fastart PCR Master, 2 µL of each primer (0.4 µM), 16 μL of RNase-free water, and 5 µl of DNA. Amplified fragments were visualized by electrophoresis on a 0.8% agarose gel containing 0.5x GelRed Nucleic Acid Gel staining (Biotium, Fremont, CA, USA). Bands of expected sizes were excised and purified from gel using the QIAquick Gel extraction Kit (Qiagen, Hilden, Germany) following the manufacturer’s instructions. Fragments were analyzed on a ABI 3130xl (Applied Biosystems, Carlsbad, CA, USA) and sequence analysis was done with BioEdit (version 7.2.5) software [36].

### 4.3. Multiple Alignments and Phylogenetic Analysis

All obtained nucleotide sequences in both genomic regions were aligned with the reference strains obtained from GeneBank using ClustalW included within the software MEGA 7.0 [37]. After alignment, sequences were trimmed and final fragments of 1516 bp and 1068 bp for the *gag-pol* and *pol* regions, respectively, were used for the final phylogenetic analysis. The new Belgian sequences reported in this study are available in the GeneBank under accession numbers MN784744 to MN784764 for the *gag-pol* sequences and MN784765 to MN784784 for the *pol* sequences. The phylogenetic reconstructions were performed using the neighbor-joining method implemented in MEGA 7.0 with the maximum likelihood substitution model and a statistical confidence of 1000 replicates [38]. Branches with bootstrap values of 70% or higher were considered to form a separate cluster. Genetic divergence was computed with MEGA 7.0 using the p-distance model and applying default settings. The tree topologies were also confirmed with the maximum-likelihood and the Bayesian inference methods. The maximum likelihood analysis was performed in MEGA 7.0 with the Tamura-Nei model (bootstrap values of 1000 replications) and the Bayesian analysis was externally performed using MrBayes (version 3.2.2) implemented in CIPRES Science Gateway [39,40].

### 4.4. Recombination Analysis

The RDP4 software (RDP, GeneConv, Bootscan, MaxChi, Chimera, SiScan, and 3Seq) was used to perform a recombination analysis in the *gag-pol* and *pol* regions of the SRLV sequences, respectively. Seven algorithms implemented in the software analyzed whether the Belgian strains that were added to the phylogeny could be the result of a recombination between any of the reference strains that were used in this phylogenetic analysis. Default settings were applied and recombination events were considered as significant if the P value was < 1.0E^−6^ in at least 4 algorithms [41,42].

## 5. Conclusions

Our genetic characterization showed that most SRLVs strains circulating in naturally infected sheep and goats from Belgium belong to genotype A and B strains, respectively. We, however, also found clear indications for natural cross-species transmission between sheep and goats, with the presence of genotype B strains in sheep and genotype A strains in goats. The heterogeneity between SRLV strains circulating in Belgium was further emphasized by the presence of strains that do not cluster with already described strains and which we propose to be prototype B5 strains. In order to keep SRLV phylogeny as a relevant tool for the assessment of the heterogeneity between SRLV strains and to remain helpful in the development of appropriate diagnostic tests, it seems advisable that the standards proposed by Shah et al. on sequencing fragments and amplicons lengths should be more rigorously followed for addition of new strains to avoid a wild growth of new genotypes and subtypes based on short genomic sequences.

## Figures and Tables

**Table 1:** Overview of the small ruminant lentivirus (SRLV) subtypes that have been characterized and published since 2004. RT, reverse transcriptase fragment; CA, capsid; MA, matrix; SU, surface fragments; *env*, envelope gene.

Subtypes	Genomic Regions Used for the Classification	Year	Species in Which the Subtype Was Detected	Country of Origin	References
A1	*gag-pol* (1.8 kb) *pol* (1.2 kb) RT (279 bp)	2004	Sheep	Iceland	[7]
A2		2004	Sheep	North America	[7]
A3		2004	Sheep and goats	Switzerland	[7]
A4		2004	Sheep and goats	Switzerland	[7]
A5		2004	Goats	Switzerland	[7]
A6	RT (279 bp)	2004	Sheep and goats	Southern France	[7]
A7	*pol* (1.2 kb)	2004	Goats	Switzerland	[7]
A8	*gag* (684 bp)	2007	Goats	Italy	[15]
A9	*gag* (684 bp)	2007	Sheep and goats	Italy	[15]
A10	*pol* (353 bp)	2010	Goats	Italy	[16]
A11	*gag-pol* (640 bp)	2011	Sheep and goats	Italy	[17]
A12	CA (467 bp) MA(327 bp) SU (394 bp)	2012	Sheep and goats	Poland	[12]
A13			Sheep and goats	Poland	[12]
A14	*gag-pol* (1471 bp) *pol* (1025 bp)	2013	Goats	Slovenia	[18]
A15		2013	Sheep	Slovenia	[18]
A16	CA (467 bp) *env* (344 bp)	2018	Goats	Poland	[11]
A17		2018	Goats	Poland	[11]
A18 *	*gag* (576 bp)	2019	Sheep	Poland	[19]
A18 *	Full genome partial *gag* gene	2019	Goat	Italy	[20]
A19		2019	Sheep	Italy	[20]
B1	*gag-pol* (1.8 kb) *pol* (1.2 kb) RT (279 bp)	2004	Sheep and goats	U.S	[7]
B2		2004	Sheep	Switzerland	[7]
B3	*gag-pol* (1320 bp) *pol* (3201 bp) *env* (2814 bp)	2011	Sheep and goats	Italy	[21]
B4	*gag* (1187 bp)	2013	goats	Canada	[22]
C	*gag-pol* (1.8 kb) *pol* (1.2 kb) RT (279 bp)	2004	Sheep and goats	Norway	[7]
D	*pol* (1.2 kb)	2004	Sheep and goats	Switzerland	[7]
E1	*gag* (525 bp)	2010	Goats	Italy	[23]
E2	*gag* (525 bp)	2010	Goats	Italy	[23]

* Two subtypes A18 were published by two independent research groups within the same month. Olech et al. were the first group to published subtype A18.

**Table 2:** List of samples characterized in the present analysis. For each samples, the species, the characteristics of the farm they are originated from, the provinces, the Ct values and the subtypes obtained in the *gag-pol* and *pol* fragments are mentioned. Accession numbers are shown between brackets.

Animal Identification	Species	Type of Farm Activity	Mixed Herds with Sheep and Goats	Farms Size (Number of Animals)	Province (Belgium)	Ct Values Obtained in qPCR	*Gag-Pol* Subtype	Genetic Distances to the Closest Reference Strains (MVV1514/CAEV Cork) for the *Gag-Pol* Fragment *	*Pol* Subtype	Genetic Distances to the Closest Reference Strains (MVV1514/CAEV Cork) for the *Pol* Fragment *
A.3.4	Sheep	Hobby	Yes	7	Antwerp	Neg	A1 (MN784764)	17.02%	A1 (MN784784)	17.63%
H.4.2	Sheep	Hobby	No	17	Hainaut	Neg	**B1 (MN784761)**	**10.47%**	N.O	
LB.1.6	Sheep	Professional	No	65	Limburg	32.91	A1 (MN784760)	16.10%	A1 (MN784782)	16.75%
LK.3.3	Sheep	Hobby	No	7	Liege	33.25	A1 (MN784758)	15.95%	A1 (MN784779)	17.83%
LK.5.4	Sheep	Hobby	No	23	Liege	32.29	A1 (MN784757)	15.74%	A1 (MN784778)	18.12%
LK.6.3	Sheep	Professional	No	150	Liege	34.14	A1 (MN784756)	14.81%	A1 (MN784777)	17.43%
LX.2.1	Sheep	Professional	No	226	Luxembourg	29.1	A1 (MN784755)	15.17%	A1 (MN784776)	18.41%
OV.1.7	Sheep	Hobby	No	15	East Flanders	35.13	A1 (MN784754)	15.10%	A1 (MN784775)	17.43%
OV.13.1	Sheep	Hobby	No	10	East Flanders	33.08	A1 (MN784752)	14.60%	A1 (MN784774)	18.51%
OV.20.1	Sheep	Hobby	No	15	East Flanders	34.1	A1 (MN784751)	15.67%	A1 (MN784773)	16.85%
VB.1.2	Sheep	Hobby	No	12	Flemish Brabant	36.82	A1 (MN784750)	15.53%	A1 (MN784771)	18.41%
VB.3.1	Sheep	Hobby	Yes	7	Flemish Brabant	Neg	N.O ^a^		N.O	
VB.7.7	Sheep	Hobby	No info	7	Flemish Brabant	32.35	A1 (MN784748)	16.24%	A1 (MN784772)	17.43%
WV.4.3	Sheep	Hobby	No	10	West Flanders	35.28	A1 (MN784746)	16.38%	A1 (MN784767)	16.94%
WV.14.5	Sheep	Professional	No info	200	West Flanders	35.06	A1 (MN784744)	14.46%	A1 (MN784769)	17.43%
H.1.6	Goat	Hobby	Yes	10	Hainaut	40	**B1 (MN784763)**	**11.25%**	N.O	
H.2.6	Goat	Professional	No	100	Hainaut	36.78	**B1 (MN784762)**	**12.39%**	**B1 (MN784783)**	**13.22%**
LB.2.3	Goat	Hobby	No	3	Limburg	32.55	**B1 (MN784759)**	**12.25%**	**B5 (MN784781)**	**13.12%**
LK.3.1	Goat	Hobby	No	4	Liege	40	N.O		A1 (MN784780)	18.90%
OV.4.5	Goat	Hobby	Yes	15	East Flanders	38.97	N.O		N.O	
OV.7.7	Goat	Professional	No	70	East Flanders	37.42	**B1 (MN784753)**	**12.11%**	N.O	
VB.5.6	Goat	Professional	Yes	100	Flemish Brabant	36.65	**B1 (MN784749)**	**12.89%**	**B5 (MN784770)**	**13.22%**
WV.3.2	Goat	Hobby	No	3	West Flanders	36.7	**B1 (MN784747)**	**12.54%**	**B1 (MN784768)**	**13.12%**
WV.7.1	Goat	Professional	Yes	347	West Flanders	40	N.O		A1 (MN784766)	18.22%
WV.12.3	Goat	Hobby	No	10	West Flanders	34.12	**B1 (MN784745)**	**11.32%**	**B1 (MN784765)**	**12.63%**

^a^ no sequence data obtained * genotype A strains were compared to MVV1514; genotype B strains were compared to CAEV Cork.
